# Tuning Molecular Motion Enhances Intrinsic Fluorescence in Peptide Amphiphile Nanofibers

**DOI:** 10.1021/acs.biomac.4c00050

**Published:** 2024-03-20

**Authors:** Natchayaporn Sindhurattavej, Shreya Jampana, Mai Phuong Pham, Leonardo C. Romero, Anna Grace Rogers, Griffin A. Stevens, Whitney C. Fowler

**Affiliations:** † Department of Chemistry, Harvey Mudd College, Claremont, California 91711, United States; ‡ Department of Engineering, Harvey Mudd College, Claremont, California 91711, United States

## Abstract

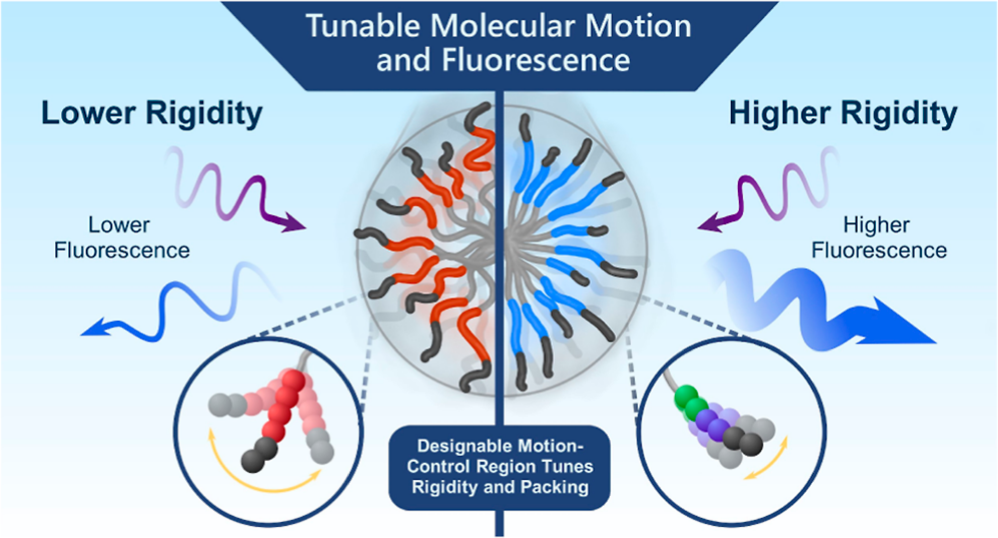

Peptide amphiphiles (PAs) are highly tunable molecules that were recently found to exhibit aggregation-induced emission (AIE) when they self-assemble into nanofibers. Here, we leverage decades of molecular design and self-assembly study of PAs to strategically tune their molecular motion within nanofibers to enhance AIE, making them a highly useful platform for applications such as sensing, bioimaging, or materials property characterization. Since AIE increases when aggregated molecules are rigidly and closely packed, we altered the four most closely packed amino acids nearest to the hydrophobic core by varying the order and composition of glycine, alanine, and valine pairs. Of the six PA designs studied, C_16_VVAAK_2_ had the highest quantum yield at 0.17, which is a more than 10-fold increase from other PA designs including the very similar C_16_AAVVK_2_, highlighting the importance of precise amino acid placement to anchor rigidity closest to the core. We also altered temperature to increase AIE. C_16_VVAAK_2_ exhibited an additional 4-fold increase in maximum fluorescence intensity when the temperature was raised from 5 to 65 °C. As the temperature increased, the secondary structure transitioned from β-sheet to random coil, indicating that further packing an already aligned molecular system makes it even more readily able to transfer energy between the electron-rich amides. This work both unveils a highly fluorescent AIE PA system design and sheds insights into the molecular orientation and packing design traits that can significantly enhance AIE in self-assembling systems.

## Introduction

Peptide amphiphiles (PAs) are a widely studied class of biomimetic molecules composed of a hydrophobic tail conjugated to a peptide headgroup that spontaneously self-assemble into ordered micellar structures in aqueous environments.^1^ Given that PAs are highly tunable on both the molecular and micro length scales and readily incorporate bioinspired functions, PAs have been designed for a wide range of applications including tunable drug release and delivery,^2^ harvest of phosphate ions for resource recovery,^3^ and *in vitro* cholesterol efflux and *in vivo* reduction of liver toxicity.^4^ Interestingly, PAs without a fluorescent tag were only recently discovered to exhibit aggregation-induced emission (AIE),^5^ a phenomenon that occurs when molecules that are non- or weakly fluorescent show a striking increase in emission efficiency upon aggregation.^6^ This finding adds an exciting intrinsic fluorescent property to the already highly useful PAs. It also unveils PAs as a desirable AIE platform, which usually are not soluble in water^7^ and are difficult to synthesize, control, and functionalize.^8^ However, the scientific community has yet to tap into decades of PA molecular design insights to optimize the fluorescence of self-assembling PAs.

Rigidity is one such material characteristic of PAs that has been thoroughly characterized and controlled through molecular design, and it also directly relates to AIE performance. AIE aggregates fluoresce as a result of spatial proximity of electron-rich moieties, such as aromatic rings and amide bonds, and restriction of intramolecular motion, causing the excess energy of the moieties to be emitted as light instead of being lost through rotational or vibrational motion.^9,10^ Pashuck *et al.* were the first to determine that incorporating various combinations of valine and alanine in the peptide headgroup area closest to the core strongly impacted the overall rigidity.^11^ More recently, Stupp and co-workers used molecular dynamics simulations to determine a strong correlation between valine–alanine–glycine combinations to the molecular motion of the PA molecules within the nanofibers.^12^ They then successfully demonstrated that molecular motion within nanofibers directly correlated to functional performance, showing that increased molecular motion enhanced nerve cell regeneration. While the rigidity of PAs has been quantified and systematically modified in previous works, this insight into molecular motion has not yet been leveraged to tune AIE properties in PAs.

Here, we present our work on how we strategically selected molecular designs to promote or quench intermolecular motion and, in turn, impact intrinsic fluorescence. We leveraged the design principles of modifying amino acid residues and position in the closely packed region nearest to the hydrophobic core to tune intermolecular motion and standardize nanofibril self-assembly. We then analyzed the absorbance, excitation, and emission properties of the systems and paired these data with secondary structure characterization to understand how intermolecular packing and ordering relate to both molecular motion as well as intrinsic fluorescence. We leveraged temperature as another design parameter to control molecular rigidity, measuring the fluorescence of each system from 5 to 65 °C, and again correlated this data with corresponding secondary structural data. Using both room temperature and temperature ramping data of fluorescence and secondary structure, we propose intriguing peptide design insights into what intermolecular components are most important to enhance this intrinsic AIE of PA assemblies.

This work is among the first to leverage decades of PA design studies to understand and harness intrinsic fluorescence in PAs, with both application-based and fundamental importance. By unlocking key design elements to significantly enhance the intrinsic fluorescence of PAs, we position this platform to be highly intriguing for protein-inspired sensing^5^ or bioimaging applications by strengthening the emissive signal to increase sensitivity and precision. This work also sheds more light on the poorly understood AIE mechanism through our systematic designs that precisely isolate intermolecular interactions and directly impact fluorescence. Finally, as we begin to correlate PA material properties with intrinsic fluorescence, AIE in PAs can be used as a marker for identifying and evaluating the extent to which various properties, such as rigidity, architecture, secondary structure, and packing, manifest in PAs.

## Materials and Methods

### Peptide Materials

PAs were purchased from GenScript USA Inc. where they were synthesized using FMOC solid-phase peptide synthesis. High-performance liquid chromatography was used by GenScript to purify the PAs to >90% purity.

### PA Nanofiber Fabrication

Lyophilized PA samples were dissolved in Milli-Q water at the desired concentrations. The samples were then heated at 70 °C for 1 h in a mechanical shaker at 300 rpm and were also briefly vortexed halfway through this heating process to further facilitate mixing, ensuring that the PAs reach the equilibrated extended nanofiber architecture. All samples were then equilibrated to room temperature prior to experimental use to avoid any temperature impacts on the fluorescence. All reported fluorescence measurements were made within 24 h of fabrication to ensure that measurements were made before any aggregation of nanofibers.

### SEM Imaging

Silicon substrates were plasma-cleaned for 3 min using a Harrick Plasma PDC-001-HP plasma cleaning system. 0.5 μL of PAs was then deposited onto each substrate, ranging from a concentration of 75 to 125 μM to achieve the best sample distribution in the images. The substrates were placed onto glass slides and placed in a vacuum oven to dry. A Cressington 108 sputter coater operating at 40 mA for 60 s was used to deposit gold sputters on the surfaces of the samples prior to imaging. All imaging was performed on a Hitachi SU-70 scanning electron microscope operated at 5.0 and 10.0 kV. ImageJ was used to digitally process the images.^13^ For each PA system, a total of 5–17 images and at least 20 nanofiber widths from those images were used to calculate the mean and standard deviation of the widths of each PA. From the same 5–17 images per system, 5–10 nanofiber lengths in each image were used to determine the maximum and minimum PA lengths.

### UV–Vis Absorbance Measurements

PA molecules of concentrations of 125, 250, 500, and 1000 μM were read in a quartz crystal glass cuvette with a path length of 10 mm. The absorbance was determined by ultraviolet–visible spectroscopy with an Agilent Cary 60 UV–vis spectrophotometer at wavelengths ranging from 800 to 200 nm. The standard data interval was set to 0.5 nm, and the average time was set to 0.1 s.

### Excitation and Emission Scans Using Fluorescence Spectroscopy

PA molecules of concentrations 25, 50, 125, 250, 500, 1000, 2000, 3000, 4000, and 5000 μM, depending on the system, were read in a quartz crystal cuvette with a path length of 10 mm. Excitation and emission scans were recorded using an Agilent Cary Eclipse fluorescence spectrometer. The excitation scans were recorded for PA samples at emission wavelengths varying between 303 and 320 nm, based on the system, and excitation values ranging from 190 to 290 nm. The emission scans were recorded at excitation wavelengths varying between 263 and 267 nm, based on the system, and emission values ranging from 295 to 600 nm. A Peltier temperature controller was used to control the sample chamber temperature of the fluorescence spectrometer, and emission scans were recorded approximately 1 min after each temperature reached the set point, which increased from 5 to 65 °C at 5 ° intervals. All measurements were taken at 600 V with a slit width of 5 nm. The autoexcitation and autoemission filters were used for all measurements, which produced a jump artifact in the spectra at the wavelength that the filter was switched. A correction factor was applied uniformly to all emission scans that averaged the difference before and after the jump.

### Circular Dichroism

PA molecules at a concentration of 50 μM were read in a quartz crystal cuvette with a 10 mm path length on a Chirascan V100 circular dichroism spectrometer. Three scans were performed for each sample from 190 to 250 nm with a 1 nm step size, and the data were averaged between scans. The averaged data were background-subtracted and converted to the mean residue ellipticity. The data were fit using BeStSel^14^ according to a minimum-energy calculation of a linear combination of basis data sets for α-helix, antiparallel left-twisted β-sheet, antiparallel relaxed β-sheet, antiparallel right-twisted β-sheet, parallel β-sheets, turn, and “other” basis data sets. “Other” in BeStSel includes various conformations such as bend, loop, and β-bridge that in other programs are categorized as “unordered.” Since the spectra of our PA systems with high degrees of “other” are strongly resemblant to the signature random coil spectra, we refer to this entire category as “Unstructured.”

## Results and Discussion

### Peptide Design and Self-Assembly Characterization

**Figure 1:**
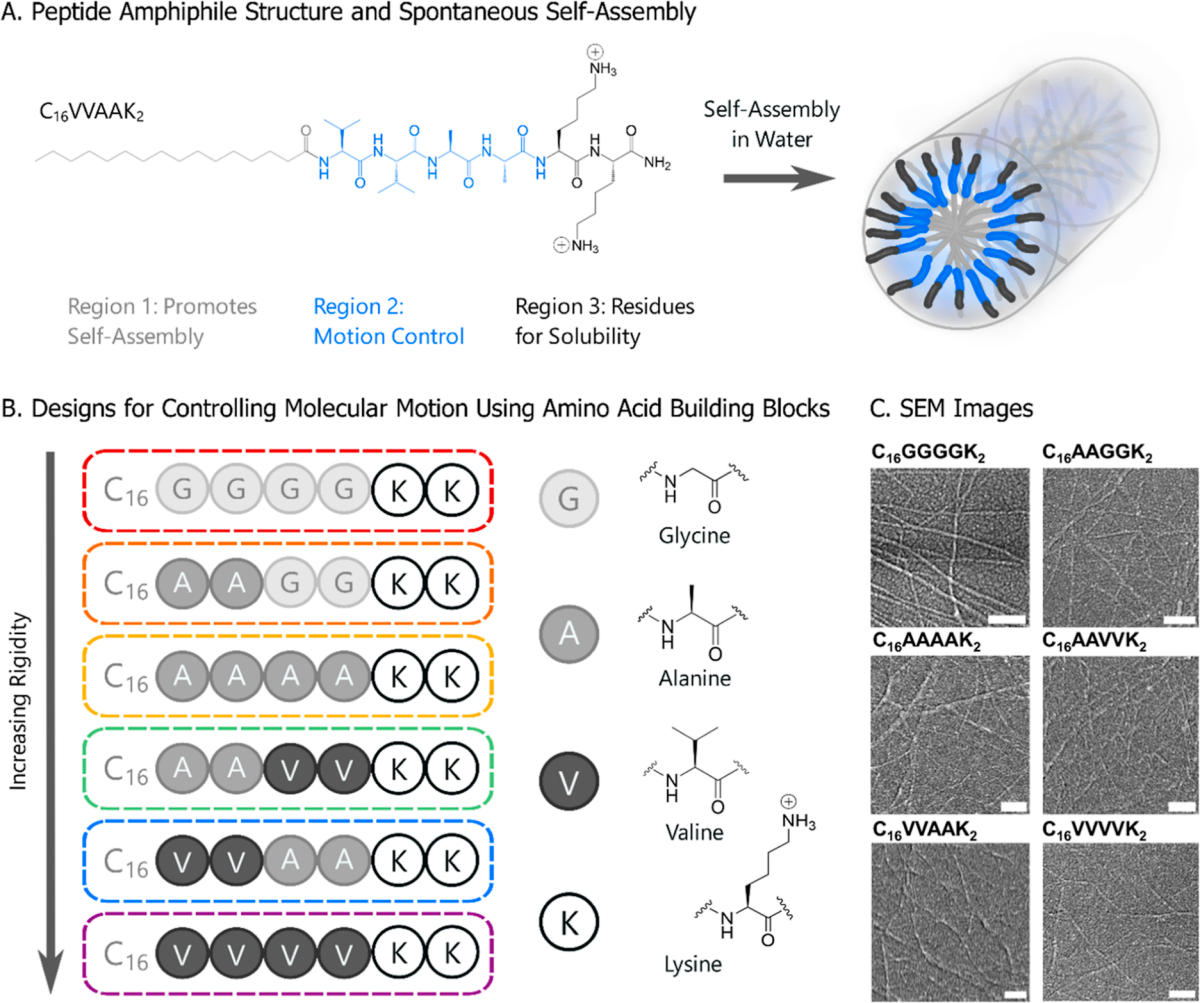
(A) Structure of a PA nanofiber using C_16_VVAAK_2_ as an example. The designed PAs have three regions: one for self-assembly, one for motion control, and one for solubility. (B) Selected PA designs composed of amino acid building blocks, namely, glycine, alanine, valine, and lysine. (C) SEM images of each peptide system confirm that all designs self-assemble into nanofibers. All scale bars represent distances of 250 nm.

PAs are unique peptide materials that exhibit precision control over both the molecular sequence design and their supramolecular assemblies. This precise control positions PAs to be extremely advantageous materials to study and enhance AIE. On the atomistic level, PAs are synthesized using solid-phase peptide synthesis, resulting in monodisperse yields that allow designers to precisely control the “building block” regions of the PA molecules.^15^ These building block regions (Figure 1A) in turn play essential roles in the supramolecular assembly and overall functionality. Region 1 consists of a hydrophobic fatty acid that drives spontaneous self-assembly in water due to the hydrophobic effect. Region 2 has been utilized as a “motion control” segment that has a strong impact on rigidity, packing, self-assembled architecture, and the secondary structure that the peptides adopt within the headgroup, due to the closely packed amino acids that interact near the hydrophobic interface.^12^ Region 3 can consist of charged amino acids for solubility, which is true in this case and is commonly used for “filler” PAs in mixed PA assemblies,^16^ or it can also often be a protein-derived binding moiety to target cell receptors or ions. Region 3 of the original PA AIE discovery was a protein-derived phosphate-binding moiety.^5^ The PA fluorescence signal responded to phosphate binding, unveiling PAs as an exciting molecular-recognition platform for protein-inspired sensing. In addition to this multilength-scale tunability, PAs are water-soluble, environmentally benign, easy to functionalize, and thoroughly studied.

We hypothesized that we could increase the PA intrinsic fluorescence intensity and quantum yield (QY) by decreasing the PA molecular motion and increasing the packing within the nanofibers. These two parameters—rigidity and packing—are two of the required qualifications of AIE, which relies on electron-rich moieties to be in close proximity and exhibit reduced rotational or vibrational motion upon aggregation. It has been shown that less mobile AIE molecules exhibit higher emission because more energy is converted into emission compared to kinetics.^17^ The proposed AIE mechanism of PA nanofibers is that the amide bonds in the peptide backbone constitute enough electron density such that when they are rigidly packed in the nanofiber corona, they exhibit AIE.^5^ This unexpected AIE has also been observed in other amide-based aggregates.^18^

To design our PAs, we leveraged two decades of work that examined how amino acid placement and composition directly impact PA nanofibril rigidity and packing. Regarding amino acid placement, it has been found that the four amino acids closest to the core of the micelle exhibit the greatest control of the overall rigidity, due to their closely packed nature near the hydrophobic core.^19^ This enhanced rigidity is often due to the formation of β-sheets in this region. Regarding composition, valine, alanine, and glycine have all been used in PA designs to tune rigidity and packing,^11,12,19^ and they have all also been found to be overrepresented in rigid residue groups in native proteins.^20^ In proteins, valine is associated with rigidity more frequently than both alanine and glycine.^20^ In PA assemblies, valine has an especially high propensity to form β-sheets, which when close to the hydrophobic core is associated with an increase in rigidity, storage modulus, and mechanical stiffness of nanofiber gels.^11^ Conversely, alanine close to the hydrophobic core decreases the rigidity, gel stiffness, and storage modulus relative to valine, likely due to its propensity to form α-helices.^11^ Recently, Stupp and co-workers used molecular dynamics models to correlate how changing the four amino acids closest to the core with various sequences of glycine, alanine, and valine induced a change in molecular motion. A high degree of motion was observed in GGGG and AAGG, while VVAA showed the lowest molecular motion.^12^

We designed six different PA systems (Figure 1B) to create a spectrum of PA designs that ranged in PA motion within the self-assembled nanofibers. Holding regions 1 and 3 constant between designs, we controlled PA motion by varying the four amino acids of region 2 by exchanging pairs of glycine, alanine, and valine. The closer the β-sheet-forming amino acids were to the core, the more likely they were to interact and increase the overall rigidity of the system. Correspondingly, the second pair of amino acids was less likely to influence the system’s rigidity. Region 1 consisted of a 16-carbon hydrophobic tail, denoted as C_16_, which has commonly been used in PA designs. Region 3 consisted of two lysines, denoted as K_2_, which promoted the solubility in aqueous solutions. The six PA designs, in increasing hypothesized rigidity, were as follows: C_16_GGGGK_2_, C_16_AAGGK_2_, C_16_AAAAK_2_, C_16_AAVVK_2_, C_16_VVAAK_2_, and C_16_VVVVK_2_.

SEM images (Figures 1C and S1–S6) confirmed that each system self-assembled into nanofibers that extended micrometers in length, with an average diameter of 30 nm. This identical architecture allowed us to directly compare rigidity and packing between PA systems with the same self-assembled architecture. AIE is also likely impacted by differing degrees of packing within various micelle architectures, from spherical micelles to nanofibers to vesicles, according to the Israelachvili packing parameter of surfactant molecules.^21^ The PA systems under study eliminated that variable and instead focused on varying degrees of packing within nanofibers attributed primarily to secondary structure.

### Fluorescence Properties of PA Nanofibers

**Figure 2:**
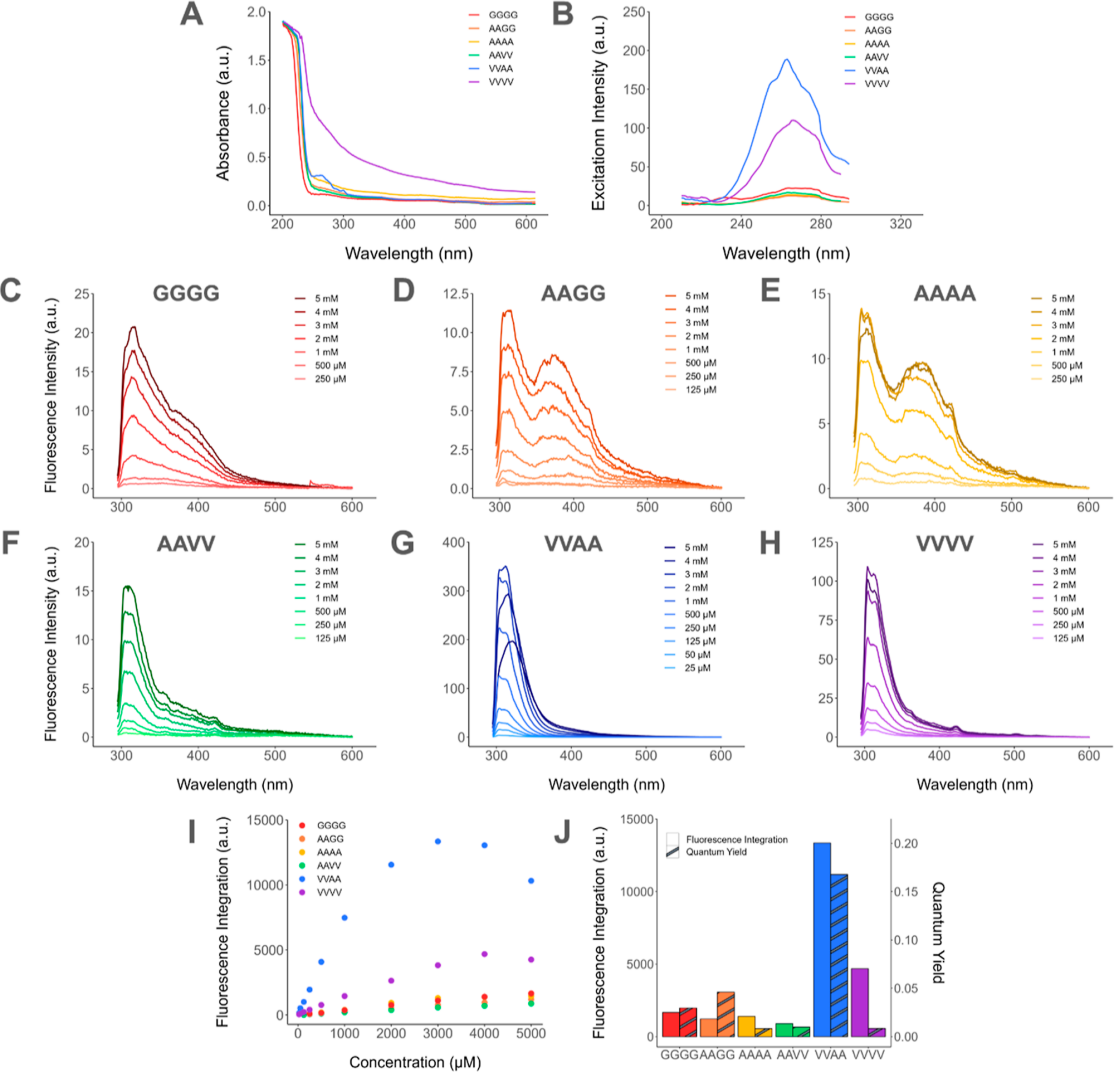
Fundamental fluorescence characterization for all six PA systems. (A) Absorbance spectra at 1 mM. (B) Excitation spectra at 5 mM at the corresponding maximum emission wavelengths. (C–H) Fluorescence emission spectra at their maximum excitation wavelengths (between 263 and 267 nm) for each of the six systems at concentrations ranging from 5 mM down to a minimum of 25 μM. (I) Integrated fluorescence emission intensity for each PA system at concentrations ranging from 125 μM to 5 mM. (J) Integrated fluorescence intensity at the concentration with maximum emission and QY of each PA system. C_16_VVAAK_2_ had both the highest fluorescence intensity and QY of all systems.

To test our hypothesis, we characterized and compared the intrinsic fluorescence properties of the six PA systems, measuring absorbance spectra, fluorescence excitation and emission spectra, integrated fluorescence emission intensity, and QYs. The results are presented in Figure 2.

First, absorbance was measured to (i) observe any unique absorbance traits that could impact the fluorescence emission or QY and (ii) determine wavelengths at which to excite the PAs. Notably in Figure 2A, C_16_VVVVK_2_ exhibited a comparatively broader peak band. Thus, this system can absorb light over a wider range of wavelengths, which also likely impacts the system’s fluorescence performance. This broad peak band could be due to C_16_VVVVK_2_ possessing additional vibrational energy levels available at each electronic energy level, or it could be that the detector is saturated at high absorbance readings.^22^ Every PA system showed the characteristic 220 nm absorbance peak for peptides, and C_16_VVAAK_2_ exhibited an additional 263 nm peak; all other PA systems showed at least a small shoulder around this wavelength as well. Using this result, the optimal excitation wavelength for all systems was found to occur between 263 and 267 nm (Figure 2B; Table S2 details the maximum wavelength for each system). At those excitation wavelengths, the maximum emission occurred between 303 and 315 nm (Figure 2C–H). These maximum excitation and emission wavelengths are lower than those of the previously studied PA AIE system, which reported an excitation wavelength of 355 nm and an emission wavelength of 430 nm.^5^ Other peptide systems exhibiting AIE also reported higher maximum excitation wavelengths, typically varying between 300 and 600 nm.^23−25^ This discovery unveils conditions under which much higher fluorescence may occur in AIE PA systems that may have been previously unexplored.

The fluorescence emission intensity results confirm our hypothesis that decreasing the molecular motion of PAs through molecular design increases the intrinsic fluorescence. C_16_VVAAK_2_ had the highest integrated fluorescence emission intensity (Figure 2J), which was the sequence reported by Stupp and co-workers to exhibit the lowest molecular motion of the sequences they studied.^12^ C_16_VVAAK_2_’s fluorescence maximum intensity was more than 3 times greater than that of C_16_VVVVK_2_ and approximately 10 times greater than those of the remaining systems. Though C_16_VVVVK_2_ also was designed to form strong β-sheets to constrain motion, its emission intensity is notably lower than that of C_16_VVAAK_2_. This is presumably due to the highly absorptive properties of C_16_VVVVK_2_. It absorbs light between 300 and 400 nm, so it likely reabsorbs the emitted light in that range, lowering the measured emission value. C_16_VVAAK_2_ is also strikingly more fluorescent than C_16_AAVVK_2_. Pashuck *et al.* studied the gel formation of PAs and found that V_3_A_3_, which has valines close to the core, is significantly more rigid than A_3_V_3_, which aligns with our hypothesis and similar system designs.^11,12^

The QYs of all six systems were calculated to normalize and further compare their emissive properties (Figure 2J). By definition, the QY is the ratio of photons emitted to photons absorbed. It was calculated by comparing the fluorescence emission intensity integration versus absorbance for each PA system at various concentrations (Figure S10) to a tryptophan standard solution, which has a QY of 0.14 (Figure S11).^26^ C_16_VVAAK_2_ had the highest QY at 0.17, which was significantly higher than those of all other systems, and an order of magnitude higher than the previous AIE PA system.^5^ C_16_AAGGK_2_ and C_16_GGGGK_2_ had the next highest QY values at 0.046 and 0.030, respectively. The QYs of C_16_AAVVK_2_, C_16_VVVVK_2_, and C_16_AAAAK_2_ were significantly lower at 0.0099, 0.0085, and 0.0083, respectively. C_16_VVAAK_2_ possessing the highest QY is once again consistent with our hypothesis that decreased molecular motion leads to increased fluorescence. C_16_VVVVK_2_ had the second-highest fluorescence emission intensity but one of the lowest QYs due to its high absorbance. This significant increase in QY for PAs, without a fluorescent tag, elevates PAs to be a highly competitive and modular platform for use as a protein-inspired sensor, where a designer can use the base of C_16_VVAAK_2_ and then easily design a protein-inspired binding moiety for region 3 to perform stimuli-responsive fluorescent signaling.

Several other important insights can be derived from Figure 2. First, additional local maxima are observed in the emission spectra (Figure 2C–H), specifically at around 375 and 425 nm. The 375 nm peak is most prominent in C_16_AAGGK_2_ and C_16_AAAAK_2_, but a shoulder is observed in all systems around this value. A smaller and distinct peak at around 425 nm is most clearly observed in C_16_VVVVK_2_ and C_16_AAVVK_2_, although it is still slightly visible for C_16_AAGGK_2_ and C_16_AAAAK_2_. The 425 nm peak likely corresponds to the reported 430 nm peak of the original PA AIE system.^5^ Second, the fluorescence emission intensity is concentration-dependent and has maximum emission intensity concentrations of 3 mM for C_16_VVAAK_2_ and 4 mM for C_16_VVVVK_2_ (Figure 2I). For all other systems, the emission intensity increased linearly as the concentration increased to the highest measured concentration of 5 mM. Shifts in the peak maxima were also observed at concentrations greater than the maximum emission for C_16_VVAAK_2_ and C_16_VVVVK_2_. This nonlinearity and concentration-dependent red shift can be attributed to the inner filter effect,^27,28^ which causes a decrease in fluorescence intensity due to the light being reabsorbed at high concentrations. Both of these insights impact how PAs may be deployed as sensors: multiple local maxima may all respond differently to peptide interactions with analytes, and deploying the PAs at their respective concentrations for the highest emission will enhance their sensing signal.

### Secondary Structure for Mechanistic Insights into Intrinsic Fluorescence

To further understand the mechanism through which the PA designs enhance or quench fluorescence, we analyzed the secondary structure that the peptides adopt in the corona of the nanofiber. The secondary structure illuminates the rigidity and packing of PAs and how their electron-conjugated sites arrange to form emission pathways among amides, which are critical variables to characterize in this AIE study. On the one hand, it is known that AIE systems emit higher fluorescence when they are more rigid and when their electron moieties are coplanar in intermolecular couplings.^29^ β-sheets lead to an ordered structure of H-bond formations, causing the PAs with higher β-sheet content to be more rigid or less mobile.^11,12^ β-sheets were also found to promote coplanar electron conjugation. While a more rigid and orderly structure is potentially more fluorescent, it has also been observed in peptide-based AIE systems that a lack of structure can allow the peptide chains to pack with a closer proximity between subfluorophores than the 5 Å intermolecular distance of β-sheets.^5,30^ Small spacings between subfluorophores enabled space conjugation, which is a noncovalent pathway essential to AIE. Thus, our six PA designs can shed further insights into how to best balance packing and rigidity to optimize fluorescence.

**Figure 3:**
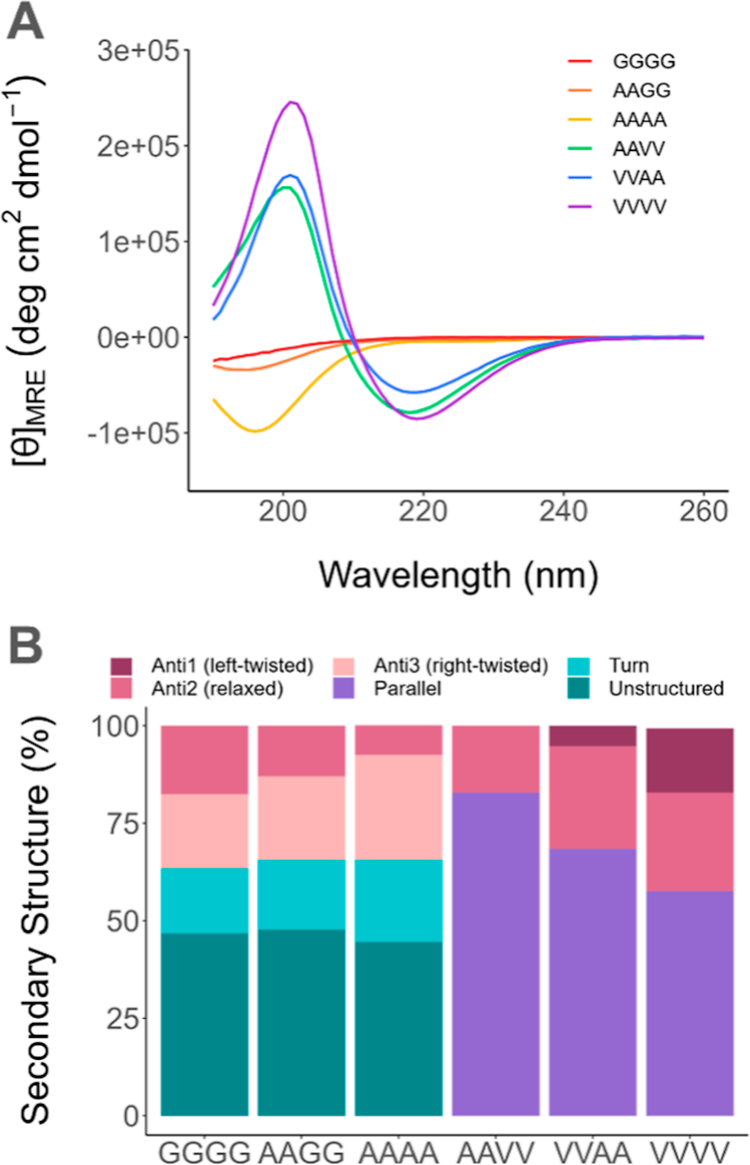
(A) CD spectra of the six PA systems acquired at room temperature. (B) The secondary structure composition of each system at room temperature was determined by BeStSel.

We acquired circular dichroism (CD) spectra to characterize the secondary structure (Figure 3A). We then fit the data with the BeStSel analysis program^14^ and present the secondary structure compositions derived from the fit in Figure 3B. BeStSel was used because we observed red shifts in the CD spectra compared to typical β-sheet spectra, and these shifts cannot be linearly corrected.^14,31^ The shifts are likely because the dielectric constant of the hydrophobic core is much higher than that of water and because the β-sheets were twisted, which was observed in previous PA nanofibers.^11^ BeStSel takes into account both the solvent effect and the sample’s twistedness, enabling us to identify the compositions of different secondary structures with a more accurate categorization of β-sheets. This categorization is informative because the hydrogen-bond directionality and handiness contribute to molecular stability.^14,32^

All six PA systems form β-sheets with various ratios and degrees of twistedness. All three valine-containing systems formed prominently parallel β-sheets and some antiparallel β-sheets of small right-handed twistedness. In contrast, the other three systems without valine adopted an unstructured primary conformation, with some right-twisted antiparallel β-sheets observed. Valine has the highest propensity to form β-sheets compared to all other natural amino acids,^11,33^ which aligns with our result that the valine-containing systems do not form any structure other than β-sheets at room temperature. Their distinctly high ratio of parallel β-sheets can be explained by the bulkiness of valine side chains, which causes the packing of branches in parallel β-sheets to become energetically favorable. In contrast, alanine is an α-helix-former.^33^ Thus, alanine tends to favor antiparallel β-sheets, which accommodate a greater degree of twist.^34^

Combining amino acid placement, secondary structure, and fluorescence performance, we propose intriguing PA design insights to understand and enhance AIE. First, a more rigid and ordered system with high β-sheet content is not necessarily more fluorescent than a system with a random coil content. C_16_AAVVK_2_ adopted 100% β-sheet conformation but had the lowest fluorescence emission of all six systems and is of the same order of magnitude as the systems with a predominantly random coil. Rather, precise amino acid placement is a much stronger factor in enhancing fluorescence. C_16_AAVVK_2_ and C_16_VVAAK_2_ have the same amino acid composition and both adopt similar β-sheet structures, but placing the pair of valines closest to the core enhances the fluorescence 10-fold. This placement has been correlated with increasing rigidity in other PA nanofibers.^19^ According to Paramonov *et al.*, hydrogen bonds formed by amino acids closer to PA’s hydrophobic core have a greater impact on the PAs’ rigidity.^19^ Meanwhile, hydrogen bonds closer to region 3 can be easily altered by the charged moieties there, adopting a different hydrogen bond structure compared to those formed by the same type of amino acid near the core. This disruption of hydrogen bonds in the second pair of amino acids in region 2 also likely applies to C_16_VVVVK_2_ and could be another reason for its reduced emission, in addition to the system’s likely reabsorption. Thus, while the secondary structure is not the final determining factor of fluorescence, the highly directional hydrogen bonding of β-sheets can be expertly designed to enhance coplanar electron conjugation and rigidity to achieve optimized AIE.

### Harnessing Temperature to Further Tune AIE in PA Nanofibers

In addition to altering the PA design to enhance AIE, we identified the potential of using temperature to control molecular mobility and arrangements of space-conjugated amides. Fluorescence of AIE systems competes with the kinetic energy which increases with molecular mobility, specifically vibration and rotation.^17^ As the temperature increases, the molecule’s electronic, vibrational, and rotational high-energy states all become more populated. However, the quantized states involving kinetics have small energy gaps, so they are more significantly impacted by the temperature change than the electronic state. Thus, it was initially hypothesized that lower temperatures cause the mobility to reduce and that more energy could be dedicated to fluorescence. However, molecular mobility can be affected not only by variations in temperature itself but also by a temperature-induced transition in secondary structures.^11^ Therefore, it is expected that the fluorescence of each system will uniquely respond to the temperature.

**Figure 4:**
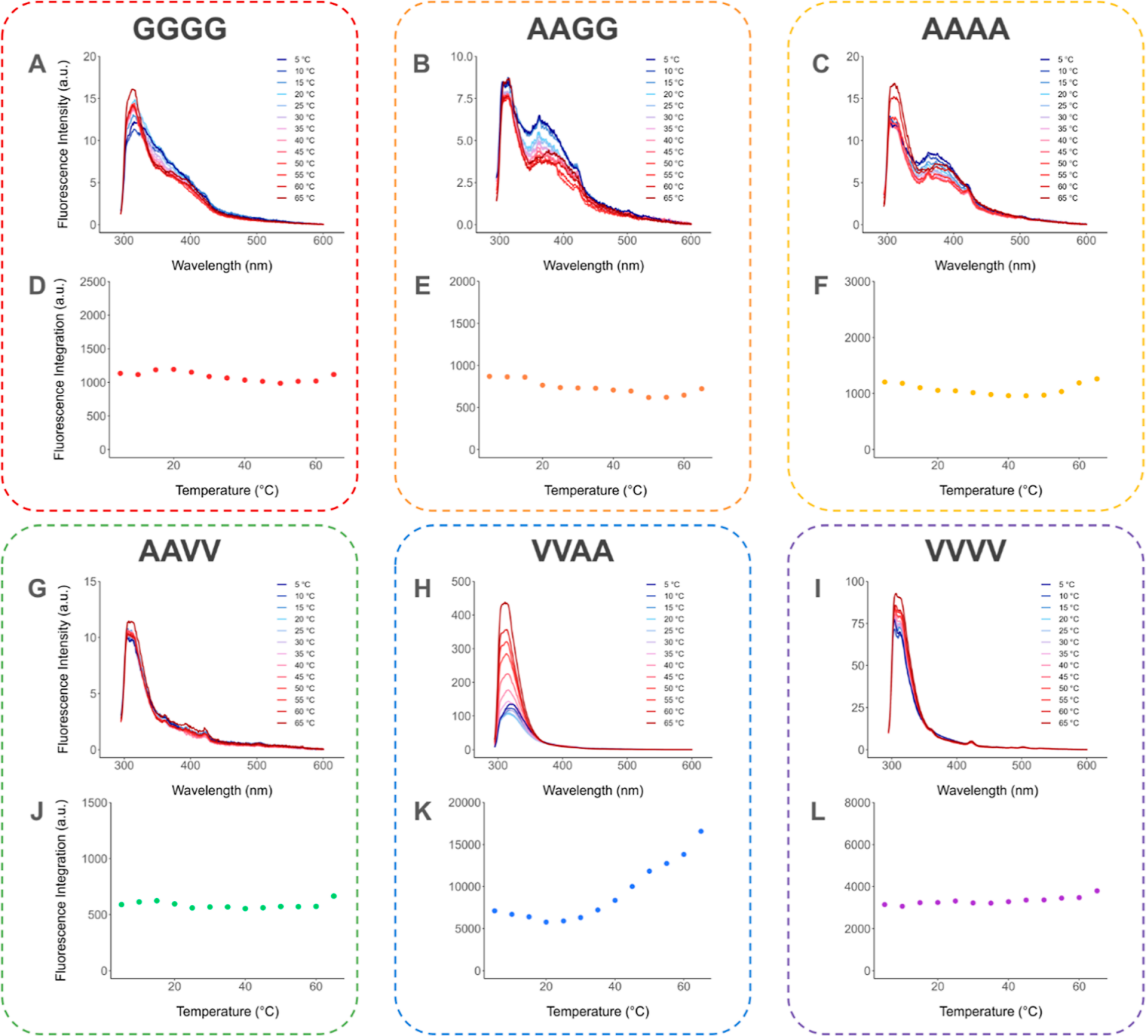
(A–C,G–I) Impact of the *in situ* temperature on the emission spectra of the six PA systems increased from 5 °C (blue) to 65 °C (red). (D–F,J–L) Integrated fluorescence emission intensities of the six PA systems to better visualize temperature-dependent trends. The fluorescence intensity of C_16_VVAAK_2_ increased 4-fold as temperature increased.

We determined how the fluorescence of each of our six PA systems changed as we increased the *in situ* temperature from 5 to 65 °C at increments of 5 °C, using the fluorometer’s Peltier. We measured each PA system at 3 mM to avoid the inner filter effect found at higher concentrations. In Figure 4, we present both the emission spectra for each temperature and the trend between the fluorescence integration and *in situ* temperature to best visualize the temperature-dependent trend of each system.

C_16_VVAAK_2_ exhibited a surprising and very significant response to the temperature (Figure 4H,K). While the other five systems were comparatively stable with temperature, the fluorescence emission intensity of C_16_VVAAK_2_ increased by more than 4 times from 5 to 65 °C. Initially, from 5 to 25 °C, the emission intensity of C_16_VVAAK_2_ decreased as the temperature increased, which aligned with our initial hypothesis that decreased temperature would correspond to decreased motion and increased AIE. However, at temperatures higher than 25 °C, the emission intensity of C_16_VVAAK_2_ began to significantly increase with temperature. The trend of emission intensity in response to temperature changes was variable for the remaining systems. From 5 to 60 °C, the emission of C_16_AAAAK_2_ and C_16_AAGGK_2_ gradually decreased while the emission of C_16_GGGGK_2_, C_16_AAVVK_2_, and C_16_VVVVK_2_ remained essentially constant. At 65 °C, all six systems started to have an increased emission or the increasing trend became even more significant.

**Figure 5:**
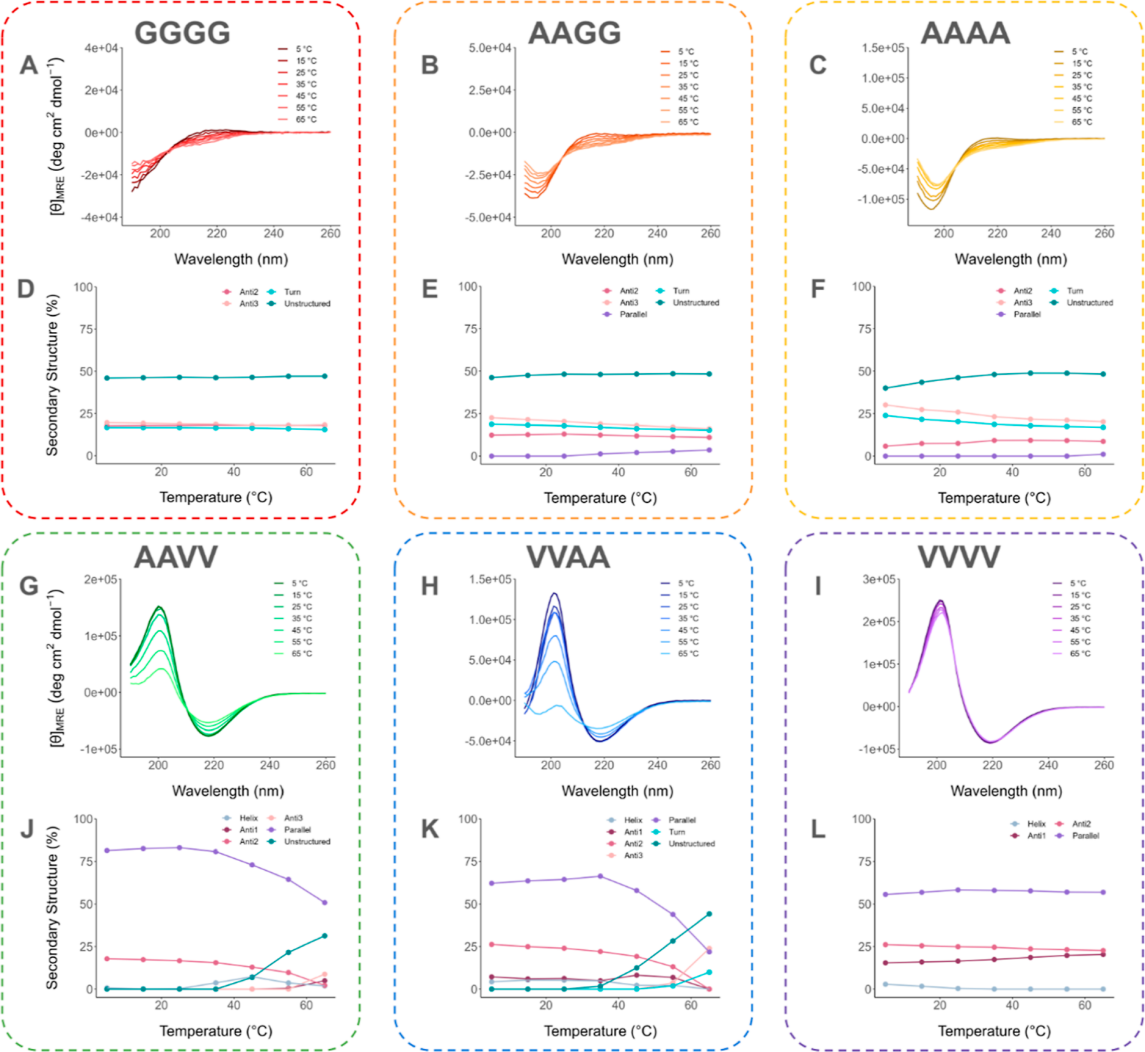
(A–C,G–I) Impact of the *in situ* temperature on CD spectra of PA systems. (D–F,J–L) Plots of the secondary structure’s composition analyzed from the spectra by BeStSel.^14^ The legends Anti1, Anti2, and Anti3, respectively, represent left-hand twisted β-sheets, slightly right-hand twisted β-sheets, and right-hand twisted β-sheets.

To interpret these results, we utilized secondary structure data derived from temperature-dependent CD spectra and paired them with the fluorescence data. In Figure 5, we present the CD spectra of each system from 5 to 65 °C at 10 °C increments, as well as a plot of the BeStSel^14^ fit for each PA system in relation to temperature.

C_16_VVAAK_2_ and C_16_AAVVK_2_ were the only systems that showed a significant shift in secondary structure as the temperature increased (Figure 5J,K). At lower temperatures, both structures initially contained only β-sheets, whereas no “unstructured” was identified. At 35 °C, they started to incorporate more “unstructured” as the overall β-sheet content correspondingly decreased. By 65 °C, the “unstructured” in C_16_VVAAK_2_ and C_16_AAVVK_2_ had increased to 31 and 44%, respectively. We speculate that this is because alanine is a helix-former. Thus, it facilitates the incorporation of turns and other unstructured conformations (random coils and loops) into the β-sheet segments, of which formation is mainly influenced by valines. Loops and turns can connect and add flexibility to the β-strands. This flexibility allows the peptide to fold more properly in a three-dimensional space, in adjustment to environmental conditions such as temperature. Based on this finding, we recognized the tunability in the secondary structure of these systems that incorporate both valines and alanines. Interestingly, turns were not identified in C_16_AAVVK_2_. Meanwhile, C_16_VVAAK_2_ is the only system in which both “unstructured” and “turn” increase with temperature. Therefore, a change in the ratio of turns could become an essential factor in the link between secondary structures and the fluorescence of a PA system since it may be a physical explanation for why the fluorescence C_16_VVAAK_2_ is the most responsive to temperature.

A similar trend of lessening β-sheets at higher temperatures was observed in a previous study, reporting that PA nanofibers transition their secondary structure from β-sheets at room temperature to random coils at higher temperatures. A few hours of recooling were required to reform an original structure.^35^ Despite this trend not applying to all PA systems, it emphasizes the importance of *in situ* temperature on the instantaneous structure of PAs. In C_16_AAVVK_2_, the parallel β-sheets continuously diminished from 25 to 65 °C. Meanwhile, the parallel β-sheets in C_16_VVAAK_2_ increased slightly from 5 to 35 °C then significantly reduced at temperatures higher than 35 °C. From 60 to 65 °C, the reduction in the parallel β-sheets is even more pronounced (from 44 to 22%), whereas the “unstructured” rapidly increases (from 28 to 44%).

In each system, the trend of secondary structures was compared with the trend of temperature-dependent fluorescence. In C_16_VVAAK_2_, we found a strong correlation between these trends at higher temperatures. From 5 °C to ambient temperature, the fluorescence of C_16_VVAAK_2_ slightly decreased, while the overall secondary structures remained unchanged. As temperature increased from the ambient temperature to 65 °C, the fluorescence then rapidly increased with a significant drop in parallel β-sheets but a rise in random coils. A slight decrease in fluorescence at the low range of temperatures was likely due to a lower molecular motion induced by lower temperatures, which then increased as temperature increased to room temperature. The fluorescence in this temperature region was likely unaffected by secondary structures which remained unchanged in this range. However, at higher temperatures, an increased content of disordered structures corresponded to an increased AIE effect for C_16_VVAAK_2_. It is remarked that the critical temperature at which the conformations started to change (35 °C) does not perfectly match that of the fluorescence trend (25 °C). At higher temperatures for C_16_VVAAK_2_, the random coil’s flexibility could promote higher molecular mobility, potentially quenching AIE. However, here we propose that this flexibility allows the electron-rich moieties to more closely pack from an already ordered β-sheet structure that is present at room temperature, optimizing packing and further enhancing AIE in a system initially rich in β-sheets. This closer packing in combination with the system’s highly rigid nature produces a hyperfluorescent system of C_16_VVAAK_2_ at high temperatures.

Interestingly, we do not observe a strong increase in the fluorescence of C_16_AAVVK_2_ as it also transitions from β-sheet to random coil when the temperature increases. We speculate that the lower rigidity of C_16_AAVVK_2_ leads to a more responsive increase in molecular motion with respect to temperature, compared to C_16_VVAAK_2_’s response. Concurrently, a closer packing in C_16_AAVVK_2_ is less significantly influenced by an increased temperature, compared to C_16_VVAAK_2_. This is based on the assumption that a shift from β-sheet to random coil mainly occurs in a peripheral peptide region. In the PA nanofibers studied by Paramonov *et al.*, the interior amino acids form H-bonds resembling β-sheet interactions, whereas the peripheral amino acids are disordered.^19^ In our systems, we expected the outer valines of C_16_AAVVK_2_ to have steric hindrances in branch packing and they were not able to vary its degree of packing as much as alanines could in C_16_VVAAK_2_. Therefore, the trend of C_16_AAVVK_2_’s fluorescence due to increased molecular motion offsets the trend due to closer packing, resulting in its constant fluorescence throughout temperatures. This finding emphasizes that the placement of the valine close to the core is once again a crucial factor in maximizing the fluorescence and determining the fluorescence’s response to temperature in future applications.

We also observed the correlation between secondary structure compositions and fluorescence in the other PA systems. The secondary compositions of C_16_VVVVK_2_ and C_16_GGGGK_2_ were essentially constant, correlating well with their stable fluorescence between 5 and 60 °C. However, their increases in emission intensities at 65 °C cannot yet be explained by shifts in secondary structure. C_16_AAAAK_2_ and C_16_AAGGK_2_ were already rich in random coils at lower temperatures. When heated, C_16_AAAAK_2_ incorporated slightly more “unstructured” and reduced “turn.” However, the conformational shifts are so minimal that it is inconclusive how they impact the systems’ fluorescence. Therefore, this result essentially infers that the fluorescence of C_16_AAAAK_2_ and C_16_AAGGK_2_ decreased, in agreement with the general trend of increased molecular motion at higher temperatures.

### Insights into Peptide Design, Intermolecular Nanofibril Interactions, and Enhancing Fluorescence

Increasing rigidity enhances AIE, and increasing packing enhances AIE; here, we do both in PA nanofibers to achieve a hyperfluorescent system. Accordingly, we derive key design insights into optimizing both parameters.

A principal design insight gained from this study is that placing valine residues close to the hydrophobic core is essential to dampening molecular motion and achieving a drastic increase in fluorescence. A corollary insight is that high fluorescence is sustained when the next pair of amino acids is more flexible in nature, with the VVAA sequence exhibiting trifold greater fluorescence than the double valine-pair VVVV sequence. Phrased another way, it is critical to have β-sheet-forming components at the interface of the core and corona to give order and alignment when the amino acids are in closest proximity, and then it is equally important for the system to have less order soon after, possibly to allow for the already rigidly aligned molecules to pack even more closely. Interestingly, this pattern of alignment first and subsequent flexibility and increased packing is also observed in the intriguing temperature dependence of C_16_VVAAK_2_. Fluorescence was significantly magnified when C_16_VVAAK_2_ transitioned from the initial high rigidity of parallel β-sheets at room temperature to a less ordered and potentially even more closely packed unstructured conformation as the temperature increased to 65 °C.

Pairing these trends together, we arrive at the consummate design insight from this study: AIE is most enhanced when systems are first rigidly locked into place through β-sheet ordering and then allowed greater freedom to tightly pack from this prealigned state to achieve maximum electron-conjugation efficiency. This exciting finding can certainly be leveraged in future designs for PA systems as well as other AIE material platforms to more fully realize the potential that AIE offers to these tunable and functional systems.

## Conclusions

We designed six PA systems to strategically tune molecular motion to enhance AIE in PAs. Of the six PA systems, C_16_VVAAK_2_, a highly rigid and low-mobility PA, was the most fluorescent out of all six systems, with a QY of 0.17. Though C_16_VVAAK_2_ and C_16_AAVVK_2_ both formed predominantly β-sheets, C_16_VVAAK_2_ exhibited a fluorescence 10 times greater than that of C_16_AAVVK_2_, highlighting the importance of placing the β-sheet-forming valine residues closest to the core to maximize rigidity. C_16_VVAAK_2_ also had a fluorescence 3 times greater than that of C_16_VVVVK_2_, indicating that fluorescence is maximized when more flexible residues are closer to the charged Region 3. The differences in fluorescence observed between C_16_VVAAK_2_ and the remaining systems, and specifically the stark difference in emission intensities of C_16_VVAAK_2_ and C_16_AAVVK_2_, suggest the crucial role that amino acids and their placement play in quenching molecular motion and generating increased AIE. We also demonstrated that increasing *in situ* temperature for C_16_VVAAK_2_ from 5 to 65 °C increased its maximum fluorescence intensity 4-fold. Pairing this with secondary structural data, we found that fluorescence increased when the parallel β-sheet composition decreased while turn and random coil increased, further suggesting that fluorescence can be optimized when an already rigidly ordered system subsequently adopts greater flexibility and a higher degree of packing efficiency.

Overall, our work unveils a highly fluorescent PA design and highlights the ease with which molecular designers can tune PAs to optimize intrinsic fluorescence by leveraging the vast literature correlating PA design with material properties and functionality. The design implications proposed from this work can be readily translated to other AIE material platforms as well as used in future designs for fluorescent PA nanofibers to incorporate protein-inspired functionality for next-generation sensing and imaging.

## Supporting Information Available

Additional data including SEM images and measurements; concentration-dependent absorption spectra; excitation and emission data for all six systems; QY methods and data; CD spectra with fitted curves; and author contributions (PDF)
