# Supplementary material for: Tuning Molecular Motion Enhances Intrinsic Fluorescence in Peptide Amphiphile Nanofibers
Source: Biomacromolecules. 2024 Mar 20;25(4):2531–41. doi: 10.1021/acs.biomac.4c00050 (PMC11005007; doi:10.1021/acs.biomac.4c00050)
Supplement: Supplementary file 1 — bm4c00050_si_001.pdf [file bm4c00050_si_001.pdf]

Supporting Information

Tuning Molecular Motion Enhances Intrinsic Fluorescence in Peptide Amphiphile Nanofibers

Natchayaporn Sindhurattavej,[†] Shreya Jampana,[‡] Mai Phuong Pham,[‡] Leonardo C. Romero,[†]

Anna Grace Rogers,[†] Griffin A. Stevens,[†] Whitney C. Fowler[‡,*]

[†] Department of Chemistry, Harvey Mudd College, Claremont, California 91711, United States

[‡] Department of Engineering, Harvey Mudd College, Claremont, California 91711, United States

Keywords: Aggregation-Induced Emission, Peptide Amphiphiles, Biomimetic Material, Molecular Design, Self-Assembly

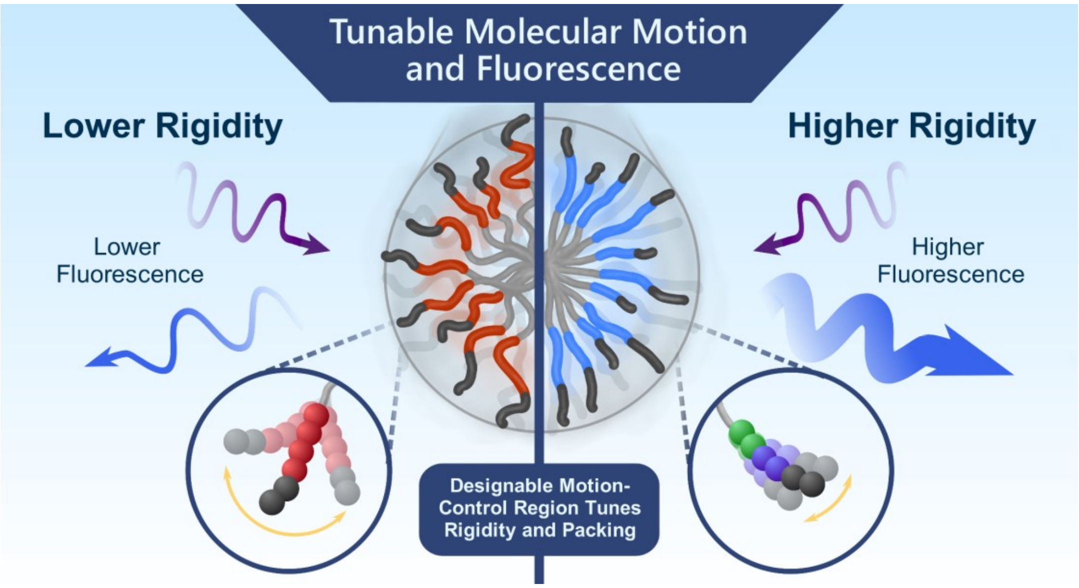

Table of Contents

1. Additional Experimental Results and Discussion
   1.1. Scanning Electron Microscopy Images S-2
   1.2. Ultraviolet-Visible Light Absorbance Spectra S-5
   1.3. Excitation and Emission Spectra S-5
   1.4. Fluorescence Emission Spectra at Different Concentrations S-6
   1.5. Quantum Yield Plots for Each System and the Tryptophan Standard S-7
   1.6. Circular Dichroism Spectra fits with BeStSel S-9
2. Author Contributions S-12

1. Additional Experimental Results and Discussion

1.1 Scanning Electron Microscopy (SEM) Images

The dimensions of Peptide Amphiphile (PA) nanofibers according to the measurement of SEM images are included in Table S1.

Table S1. Average widths and minimum and maximum lengths of nanofibers measured in each peptide amphiphile system.

PA System	Average Width (nm)	Standard Deviation (nm)	Minimum Length (nm)	Maximum Length (nm)
$C_{16}GGGGK_2$	26	7.3	727	4278
$C_{16}AAGGK_2$	29	8.4	599	4405
$C_{16}AAAAK_2$	30	4.0	1800	9882
$C_{16}AAVVK_2$	32	11	1184	7803
$C_{16}VVAAK_2$	33	9.9	985	8033
$C_{16}VVVVK_2$	25	3.4	784	3683

Additional Scanning Electron Microscopy images are included for all six PA systems at smaller magnifications to show the reader that nanofibers are present throughout the sample and to clearly show the length and width dimensions of each sample.

A

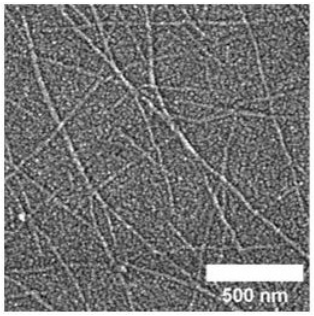

Figure S1. Additional SEM image for $C_{16}GGGGK_2$. Figure S1A is a 0.125 mM concentration. For reference, the concentration of the $C_{16}GGGGK_2$ image in the main text (Figure 1C) is 0.125 mM.

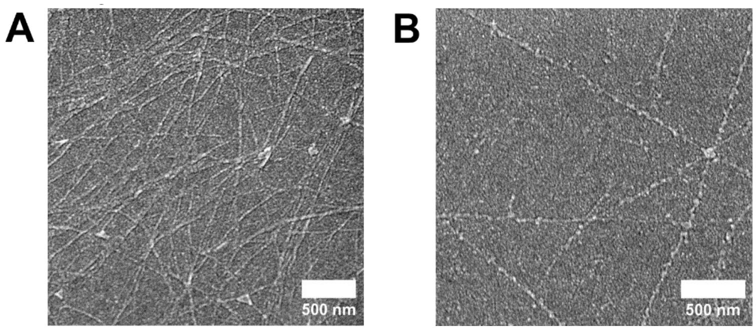

Figure S2. Additional SEM images for $C_{16}AAGGK_2$. Figure S2A is a 0.125 mM concentration, and Figure S2B is a 0.075 mM concentration. For reference, the concentration of the $C_{16}AAGGK_2$ image in the main text (Figure 1C) is 0.125 mM.

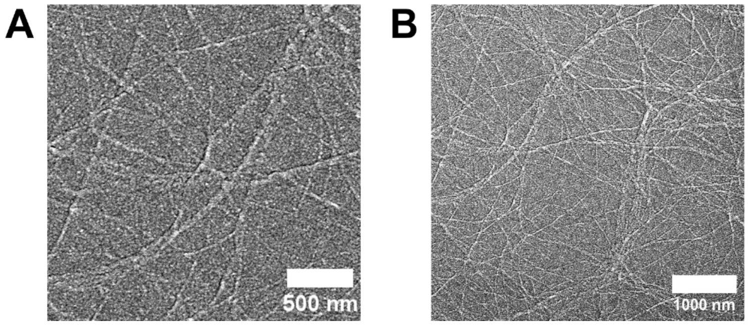

Figure S3. Additional SEM images for $C_{16}AAAAK_2$. Figure S3A is a 0.125 mM concentration, and Figure S3B is a 0.125 mM concentration. For reference, the concentration of the $C_{16}AAAAK_2$ image in the main text (Figure 1C) is 0.125 mM.

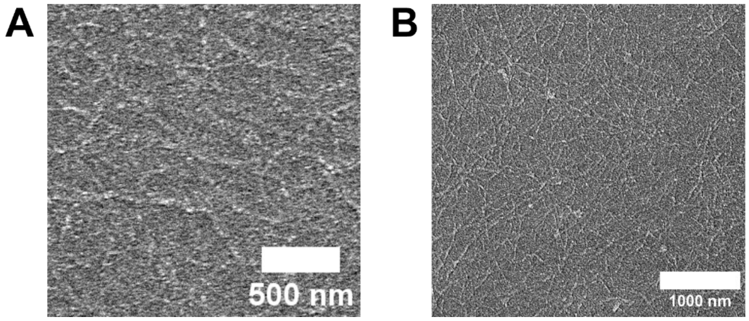

Figure S4. Additional SEM images for $C_{16}AAVVK_2$. Figure S4A is a 0.125 mM concentration, andFigure S4B is a 0.075 mM concentration. For reference, the concentration of the $C_{16}AAVVK_2$ image in the main text (Figure 1C) is 0.075 mM.

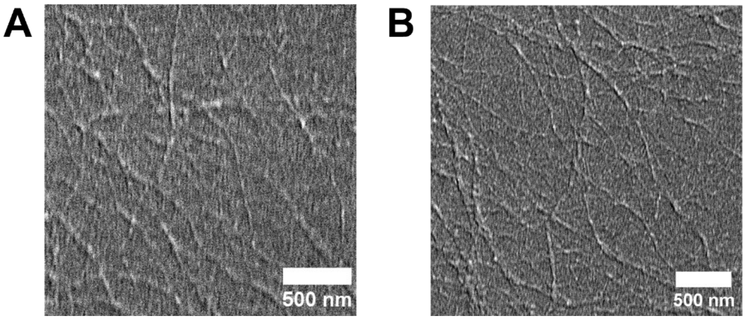

Figure S5. Additional SEM images for C_{16}VVAAK$_2$. Figure S5A is a 0.125 mM concentration, and Figure S5B is a 0.125 mM concentration. For reference, the concentration of the C_{16}VVAAK$_2$ image in the main text (Figure 1C) is 0.125 mM.

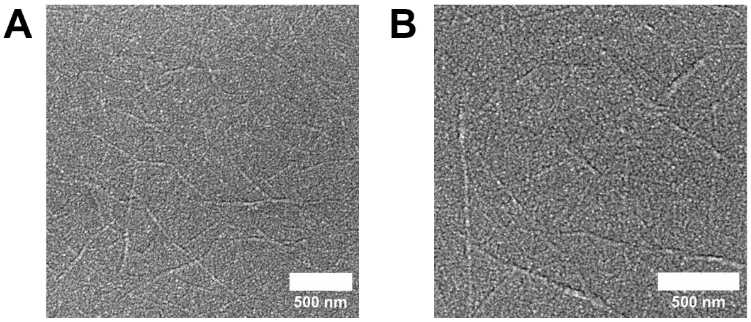

Figure S6. Additional SEM images for C_{16}VVVVK$_2$. Figure S6A is a 0.075 mM concentration, and Figure S6B is a 0.075 mM concentration. For reference, the concentration of the C_{16}VVVVK$_2$ image in the main text is (Figure 1C) 0.075 mM.

1.2 Ultraviolet-Visible Light Absorbance Spectra

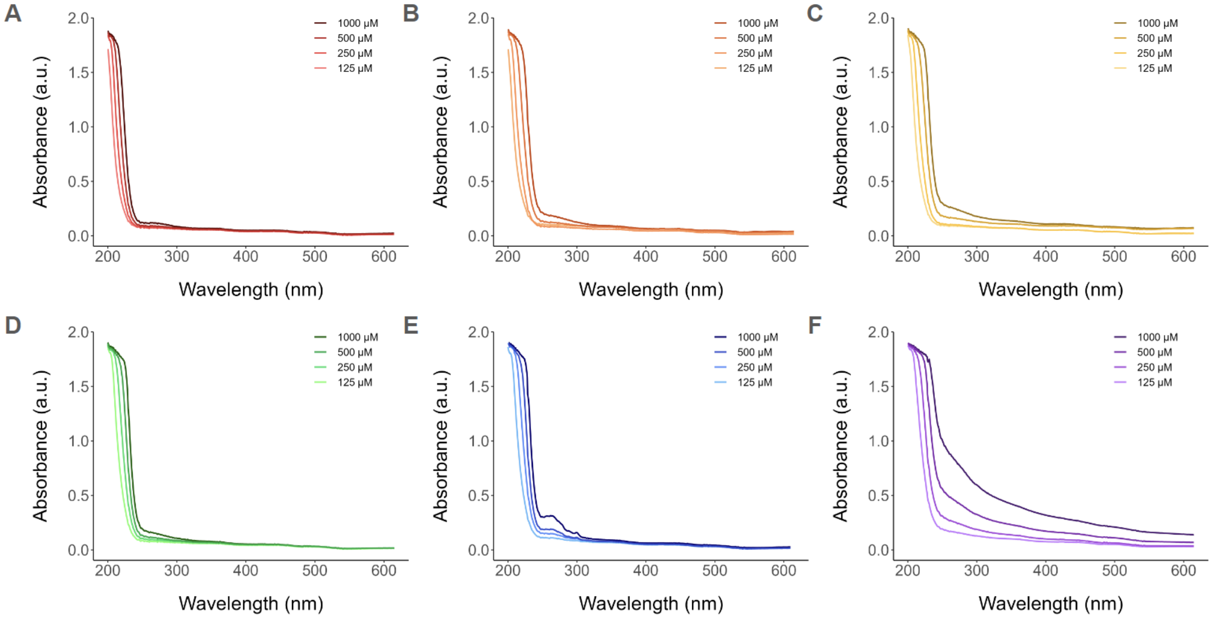

Figure S7. The absorbance spectra of the six PA systems of varied concentrations. (A) $C_{16}GGGGK_2$ (B) $C_{16}AAGGK_2$ (C) $C_{16}AAAAK_2$ (D) $C_{16}AAVVK_2$ (E) $C_{16}VVAAK_2$ (F) $C_{16}VVVVK_2$.

1.3 Excitation and Emission Spectra

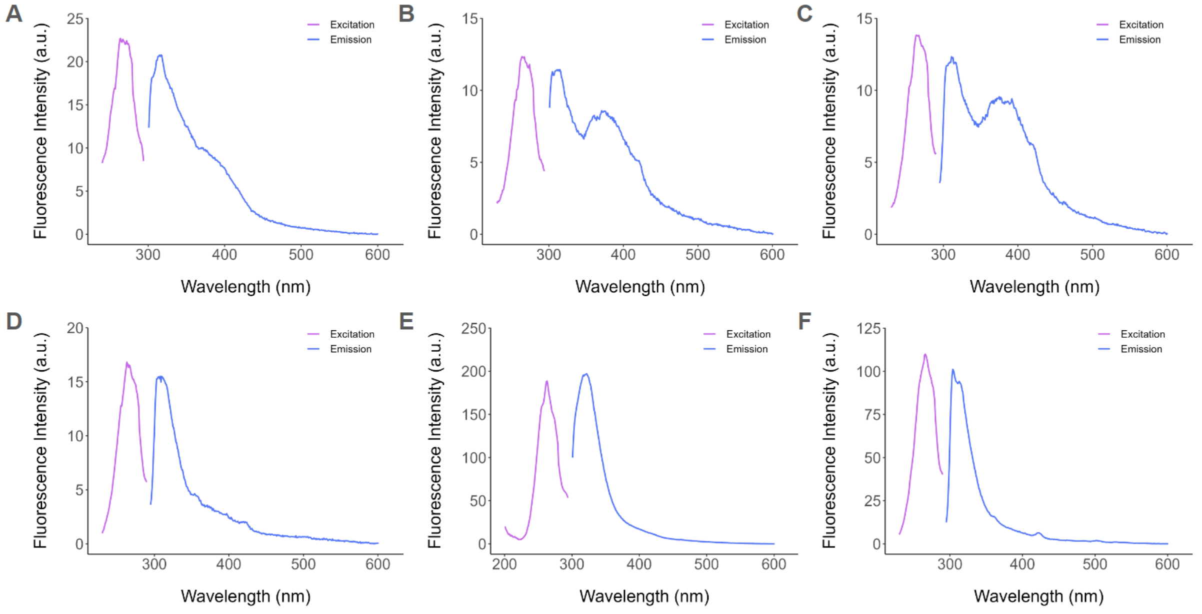

Figure S8. Overlaying excitation spectra and emission spectra of each of the six PA systems at 5 mM (A) $C_{16}GGGGK_2$ (B) $C_{16}AAGGK_2$ (C) $C_{16}AAAAK_2$ (D) $C_{16}AAVVK_2$ (E) $C_{16}VVAAK_2$ (F) $C_{16}VVVVK_2$.

1.4 Fluorescence Emission Intensity Integration with respect to Concentration

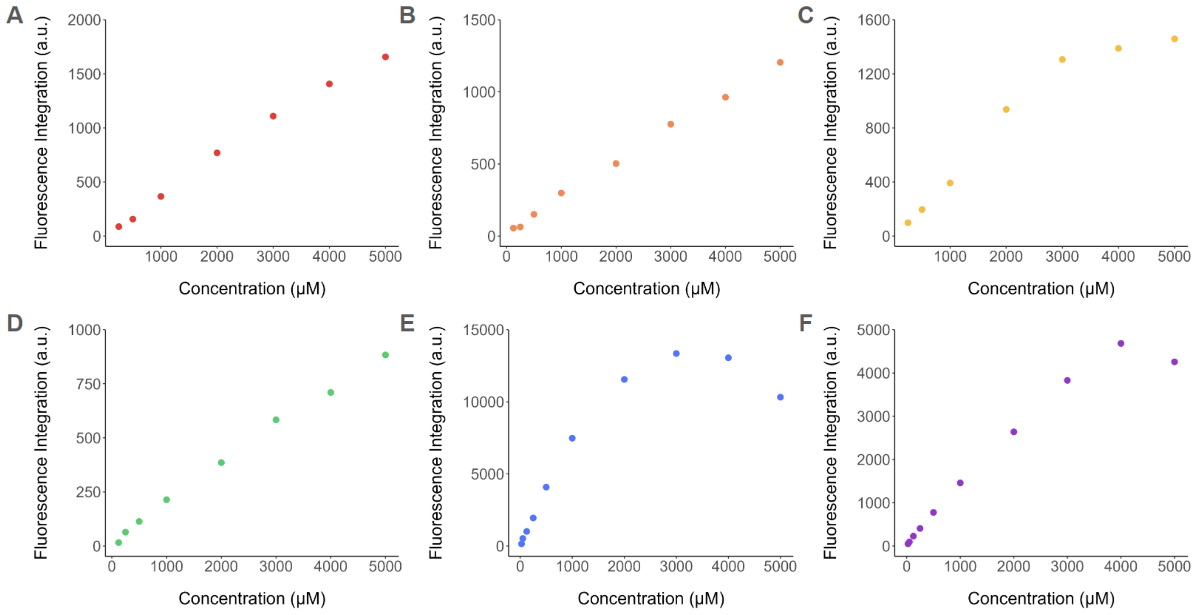

Figure S9. The relationship between fluorescence integration and concentration in each of the six systems. (A) $C_{16}GGGGK_2$ (B) $C_{16}AAGGK_2$ (C) $C_{16}AAAAK_2$ (D) $C_{16}AAVVK_2$ (E) $C_{16}VVAAK_2$ (F) $C_{16}VVVVK_2$. The fluorescence integration was calculated by integrating the area under the fluorescence emission spectrum.

Table S2. Maximum excitation and emission wavelengths at each of the PA system's respective maximum concentrations.

PA System	Concentration Producing Maximum Emission (mM)	Excitation Wavelength Used to Obtain Emission Spectra at that Concentration (nm)	Emission Wavelength Used to Obtain Excitation Spectra at that Concentration (nm)
$C_{16}GGGGK_2$	5	263.07	316.92
$C_{16}AAGGK_2$	5	264.00	314.92
$C_{16}AAAAK_2$	5	267.07	310.92
$C_{16}AAVVK_2$	5	263.07	307.96
$C_{16}VVAAK_2$	3	263.07	311.84
$C_{16}VVVVK_2$	4	266.00	303.90

Table S3. Controlled concentrations and excitation wavelengths used to test how the PA's fluorescence varies with temperature.

PA System	Excitation Wavelength (nm)
$C_{16}GGGGK_2$	263.07
$C_{16}AAGGK_2$	266.02
$C_{16}AAAAK_2$	266.00
$C_{16}AAVVK_2$	267.07
$C_{16}VVAAK_2$	263.07
$C_{16}VVVVK_2$	266.00

Each system excitation wavelength was controlled throughout temperatures, based on the maximum excitation wavelength measured at 5 °C. A concentration of 3 mM was chosen to avoid the inner filter effect.

1.5 Quantum Yield Plots for Each System and the Tryptophan Standard

The Quantum Yield (QY) for each PA sample was calculated according to the equation

$$\Phi_{PA} = \Phi_{TR}\left(\frac{Slope_{PA}}{Slope_{TR}}\right)\left(\frac{\eta_{PA}^2}{\eta_{TR}^2}\right)$$

Φ_{PA} is the QY of the peptide amphiphile. Φ_{TR} is the QY of the standard, which was tryptophan since it had a comparable emission range as the PAMs. $Slope_{PA}$ and $Slope_{TR}$ are slopes of linear plots between the fluorescence integration and the absorbance intensities, respectively for the PAMs and tryptophan. For each system, the slopes used to calculate QY were both acquired from the measurements excited by the same wavelength. η_{PA} and η_{TR} are the refractive indices for the solvents of PA samples and tryptophan, respectively.

Fluorescence emission intensity integration and absorbance intensities used to obtain the slope were samples in the linear regime (Figure S10). The emission spectra of all samples were measured by the same fluorometer using the same parameter inputs. The referenced $\Phi_{TR} = 0.14$, and the referenced $\eta_{PA} = \eta_{TR} = 1.33$.

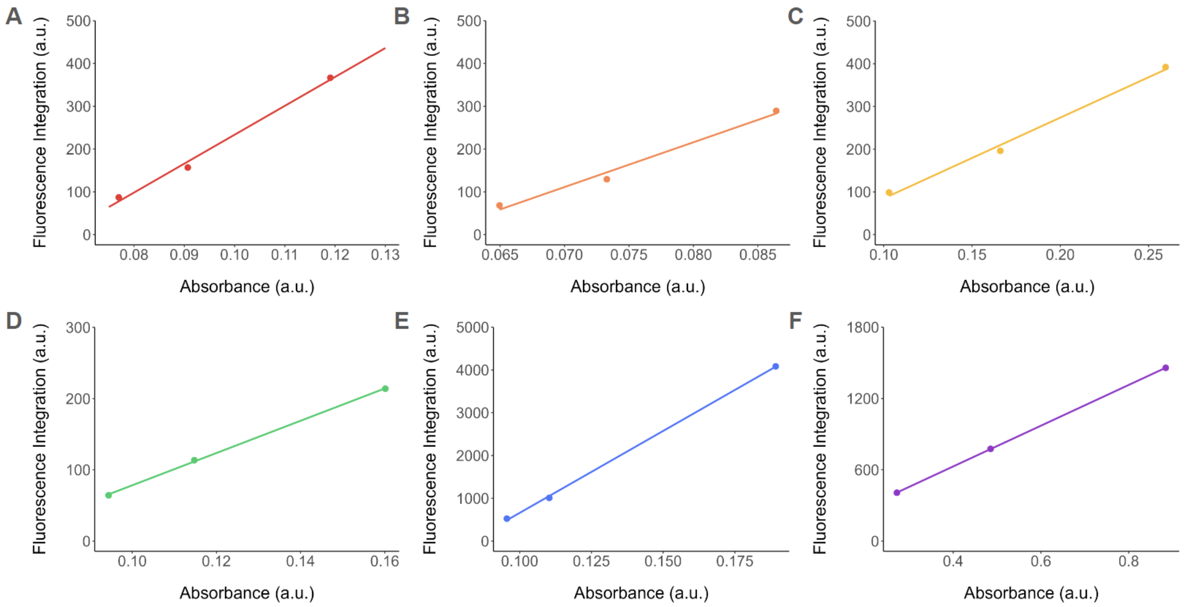

Figure S10. Quantum yield measurements show linear correlations for fluorescence emission intensity integration and absorbance intensities for all six PA systems. For $C_{16}VVVK_2$, the excitation wavelength was 266 nm while the other five PA systems used the excitation wavelength of 263.07 nm.

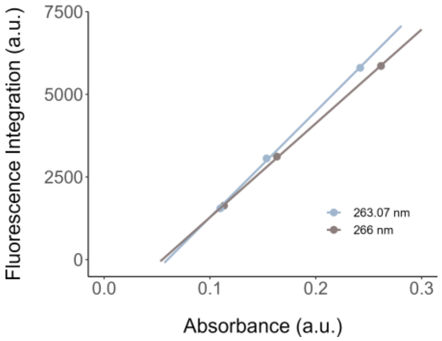

Figure S11. Quantum yield measurements show linear correlations for fluorescence integration and absorbance intensities for tryptophan. The $Slope_{TR}$ used to calculate the QY of $C_{16}VVVK_2$ was from measurements excited by 266-nm wavelength. The $Slope_{TR}$ used to calculate the other five systems was from measurements excited by 263.07-nm wavelength.

1.6 Circular Dichroism Spectra Fits with BeStSel

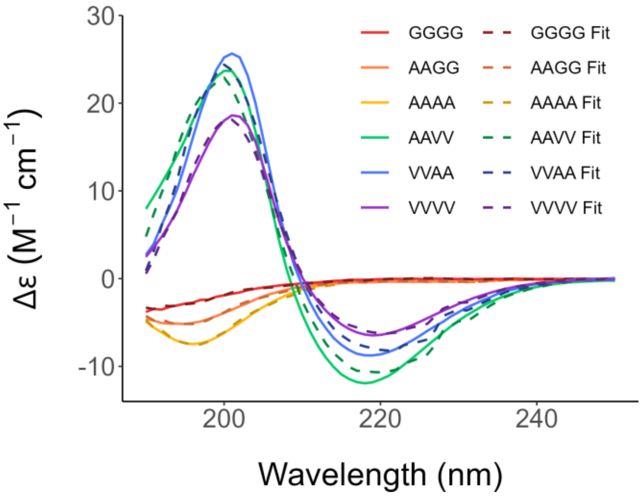

Figure S12. Room temperature CD spectra with the BeStSel fit. Shows β-sheet content in VVAA, VVVV, and AAVV while it shows random coil and unordered in GGGG, AAGG, and AAAA.

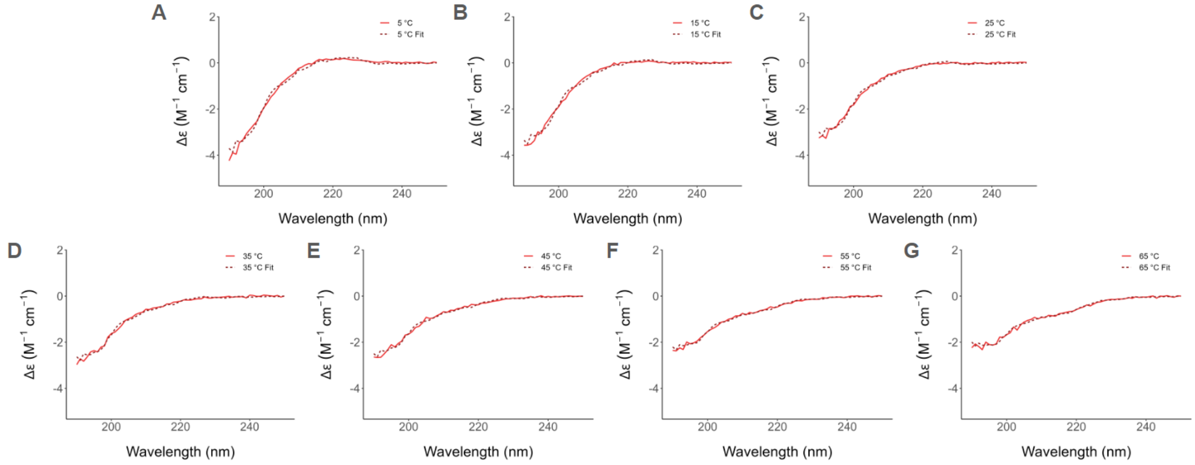

Figure S13. GGGG CD spectra fit using BeStSel at (A) 5, (B) 15, (C) 25, (D) 35, (E) 45, (F) 55, (G) 65 °C.

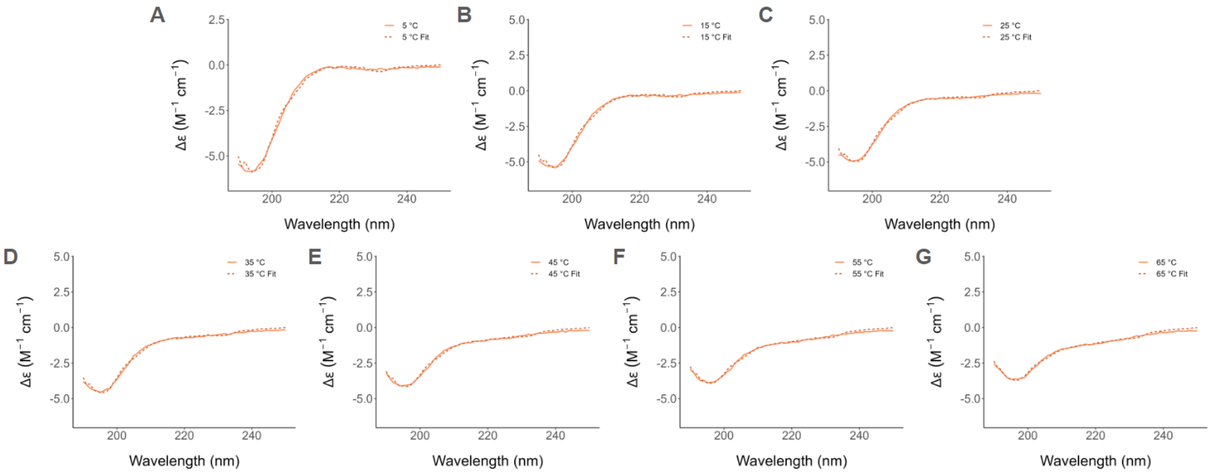

Figure S14. AAGG CD spectra fit using BeStSel at (A) 5, (B) 15, (C) 25, (D) 35, (E) 45, (F) 55, (G) 65 °C.

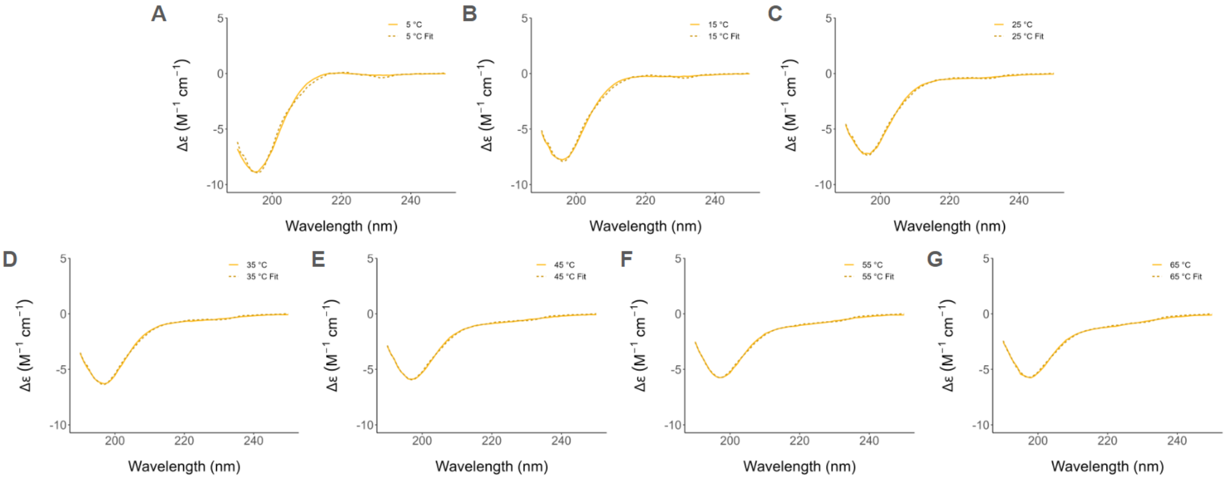

Figure S15. AAAA CD spectra fit using BeStSel at (A) 5, (B) 15, (C) 25, (D) 35, (E) 45, (F) 55, (G) 65 °C.

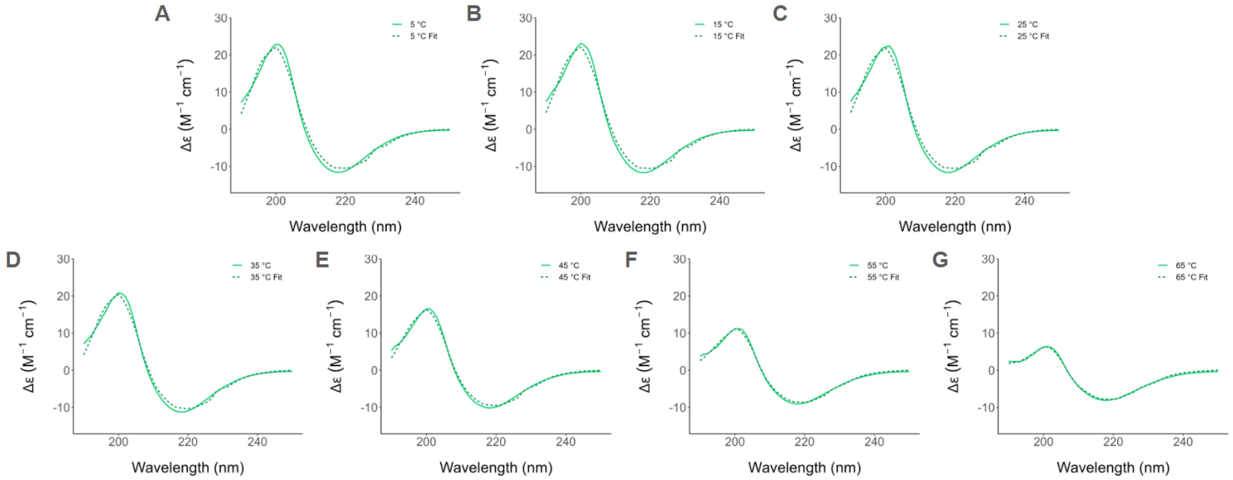

Figure S16. AAVV CD spectra fit using BeStSelat (A) 5, (B) 15, (C) 25, (D) 35, (E) 45, (F) 55, (G) 65 °C. Shows a decrease in β-sheet content and an increase in unordered.

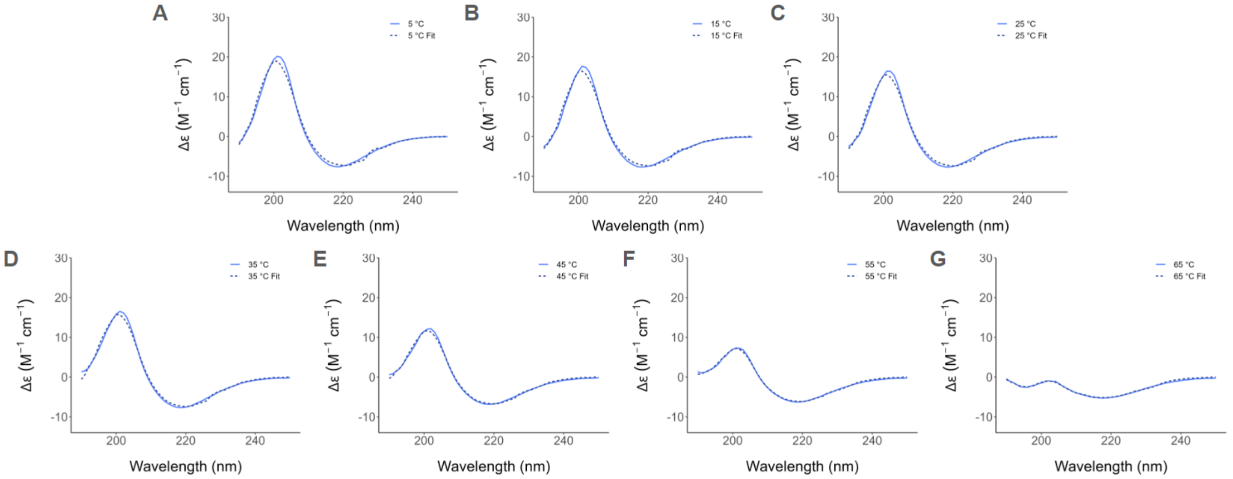

Figure S17. VVAA CD spectra fit using BeStSel at (A) 5, (B) 15, (C) 25, (D) 35, (E) 45, (F) 55, (G) 65 °C. Shows a decrease in β-sheet content and an increase in unordered/other.

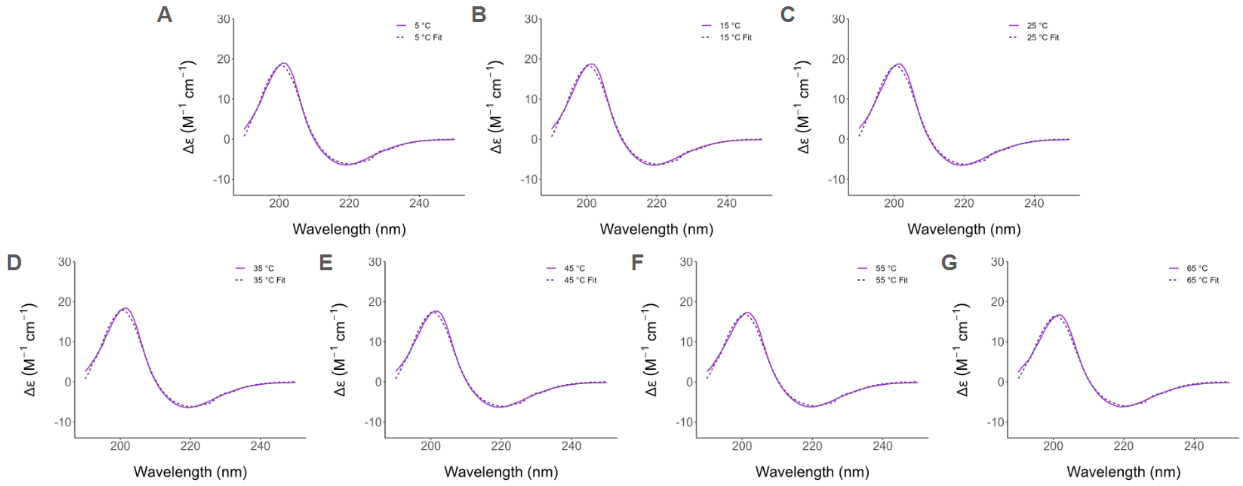

Figure S18. VVVV CD spectra fit using BeStSel at (A) 5, (B) 15, (C) 25, (D) 35, (E) 45, (F) 55, (G) 65 °C. Essentially no change in any content occurs.

2. Author Roles and Responsibilities

All authors have read and approved the completed manuscript, and all authors contributed to the PA molecular design. N.S. and S.J acquired and analyzed the fluorescence experiments and data, and they both analyzed the quantum yield data. N.S. led the writing and interpretation of the room temperature fluorescence, CD sections, and temperature-dependent fluorescence sections. S.J. contributed to the writing of the room temperature fluorescent section. M.P.P. acquired and analyzed the SEM images, contributed to the writing of the introduction and the peptide design sections, and managed all manuscript citations. L.C.R. acquired and analyzed the UV-Vis absorbance data, and he contributed to the writing of the introduction and peptide design sections. A.G.R. created all the figures in the manuscript as well as the TOC graphic. G.A.S. acquired the UV-Vis absorbance data, analyzed the CD data, and contributed to the writing of the temperature-dependent fluorescence section. The corresponding author W.C.F. oversaw the manuscript direction, management, writing, and submission as the principal investigator, and she fit the CD data using BeStSel.
